# Double Superior Vena Cava and Coexisting Vascular Variants: A Case Report and Review of Clinical Implications

**DOI:** 10.7759/cureus.99531

**Published:** 2025-12-18

**Authors:** Daniel Deschênes, William Mayer

**Affiliations:** 1 Anatomy, Dalhousie Medicine New Brunswick, Saint John, CAN

**Keywords:** azygos system variation, central venous catheterization (cvc), congenital vascular anomaly, double superior vena cava (dsvc), external jugular vein fenestration, myocardial bridge (mb)

## Abstract

A double superior vena cava is a rare congenital vascular variation that may influence cardiovascular function and medical procedures. This report describes the morphology of a double superior vena cava identified in the body of an 87-year-old female donor. Anatomical dissection revealed two fully isolated superior venae cavae, with the right superior vena cava draining into the right atrium and the left superior vena cava draining into the coronary sinus. The brachiocephalic veins were absent, and the azygos system displayed an atypical configuration. The left external jugular vein also showed significant variation, featuring a superiorly located fenestration followed by a bifurcation that formed a venous ring encircling the clavicle. The anterior interventricular artery tunnelled into the myocardium, forming two myocardial bridges along the anterior interventricular sulcus before reaching the cardiac apex. These findings illustrate the diversity of vascular anatomy and emphasize the importance of recognizing such variants during diagnostic imaging and medical procedures.

## Introduction

During embryonic development, the most caudal portion of the heart tube, the sinus venosus, bulges bilaterally, forming right and left sinus horns. The anterior and posterior cardinal veins unite to form a common cardinal vein, which drains into the lateral aspect of its respective sinus horn. As development progresses, the anterior cardinal veins anastomose transversely, forming the left brachiocephalic vein. This vein allows blood from the left side of the head and left upper limb to be funnelled mainly to the right anterior cardinal vein, whose proximal segment, together with the right common cardinal vein, ultimately forms the superior vena cava (SVC) [1]. This is followed by the regression of the left anterior cardinal vein, a process which forms a fibrous attachment known as the ligament of Marshall [2,3]. Most of the posterior cardinal veins become obliterated; however, the terminal portion of the left posterior cardinal vein is retained as the left superior intercostal vein (LSIV). This regression requires the supracardinal veins to drain the thoracic body wall. The right supracardinal vein and a segment of the posterior cardinal vein later form the azygos vein. On the left, the supracardinal vein develops into the hemiazygos vein, receiving some left intercostal tributaries before joining and draining into the azygos vein. The left horn of the sinus venosus largely regresses, with the persistent remnants differentiating into the coronary sinus (CS) and the oblique vein of the left atrium.

In the case of a double superior vena cava (DSVC), the left brachiocephalic vein can fail to form, and the left anterior cardinal vein persists, hence its alternative designation as a “persistent superior left vena cava”. In contrast to forming the ligament of Marshall during normal embryological development, a DSVC results when the left anterior cardinal vein fails to regress, resulting in direct drainage into the CS, which ultimately empties into the right atrium [1]. This accounts for the abnormally enlarged CS typically noted on the echocardiogram of an individual affected by this common variation of a DSVC [4]. Although most often terminating at the CS, some rare variations of the left superior vena cava (LSVC) may drain into the left atrium instead, creating a physiologically significant right-to-left shunt [5].

A DSVC is often found incidentally on cardiovascular imaging or surgery and is the most common congenital venous anomaly of the thorax. This venous anomaly can complicate procedures such as central venous cannulation (CVC), pacemaker placement, and cardiac transplantation [6].

## Case presentation

We present the case of an 87-year-old White female donor whose cause of death was recorded as advanced dementia. The individual was accepted into the Dalhousie Body Donation Program with the consent of their next of kin/legal executor, who dedicated the body to advancing medical education and research. Routine anatomy laboratory dissection of the neck, thorax, and abdomen revealed several vascular variations, including a DSVC with coexisting myocardial bridges (MBs), an atypical arrangement of the azygos system, and a duplicated left external jugular vein (EJV) with a superior fenestration and a venous ring encircling the clavicle.

Double superior vena cava and myocardial bridges

**Figure 1:**
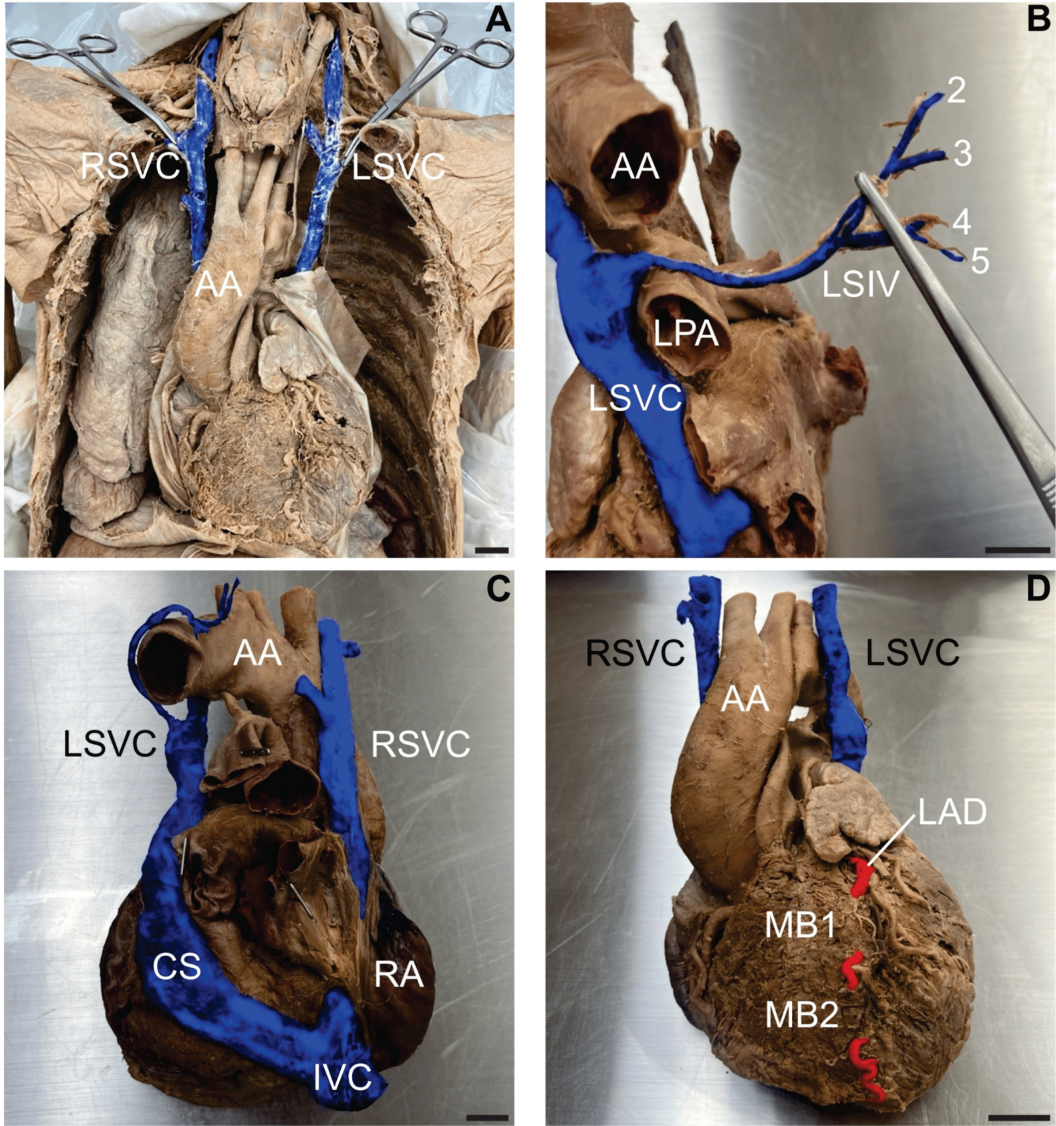
Double superior vena cava and myocardial bridges A) Anterior view of the neck and thoracic cavity demonstrating the formation of the double superior vena cava (DSVC). The right superior vena cava (RSVC) and left superior vena cava (LSVC) are each formed by the confluence of the subclavian, vertebral, and internal jugular veins. Aortic arch (AA). B) Left lateral view showing the drainage of the left superior intercostal vein (LSIV). The LSIV, formed by posterior intercostal veins 2-5, drains into the LSVC. Left pulmonary artery (LPA). C) Posterior view of the heart illustrating the DSVC drainage pattern. The LSVC drains into the coronary sinus (CS), while the RSVC drains directly into the right atrium (RA). Inferior vena cava (IVC). D) Anterior view of the heart detailing the myocardial bridges (MBs) over the left anterior descending artery (LAD). Two MBs are observed (MB 1 and MB 2). Veins are digitally coloured blue, and the artery in red. Scale bar = 2 cm.

Dissection of the superior mediastinum revealed two unusually large venous structures on each side of the aortic arch (Figure 1A). A bilateral absence of brachiocephalic veins was noted, and both venous structures were identified as fully isolated SVCs. The right superior vena cava (RSVC) drained into the right atrium, while the LSVC reached the posterior surface of the heart, draining into the CS. Both SVCs were formed by the confluence of the subclavian, vertebral, and internal jugular veins. The RSVC had a diameter of 11.80 mm and a length of 123.50 mm from its formation to the superior aspect of the right atrium. The LSVC had a diameter of 14.31 mm and a length of 84.76 mm from its formation to the entrance of the CS. The LSIV drained the left posterior intercostal spaces 2-5 and terminated within the LSVC at the level of the superior border of the left pulmonary trunk (Figure 1B). The enlarged CS measured 96.06 mm along its long axis and 25.22 mm along its short axis. The CS emptied into the right atrium (Figure 1C). Examination of the anterior surface of the heart revealed that the anterior interventricular artery, clinically known as the left anterior descending artery (LAD), coursed intramyocardially twice, forming two MBs within the interventricular sulcus before reaching the apex of the heart. The proximal myocardial bridge (MB 1) covered 17.85 mm of the LAD, while the distal bridge (MB 2) spanned 13.85 mm (Figure 1D).

Azygos venous system variation

**Figure 2:**
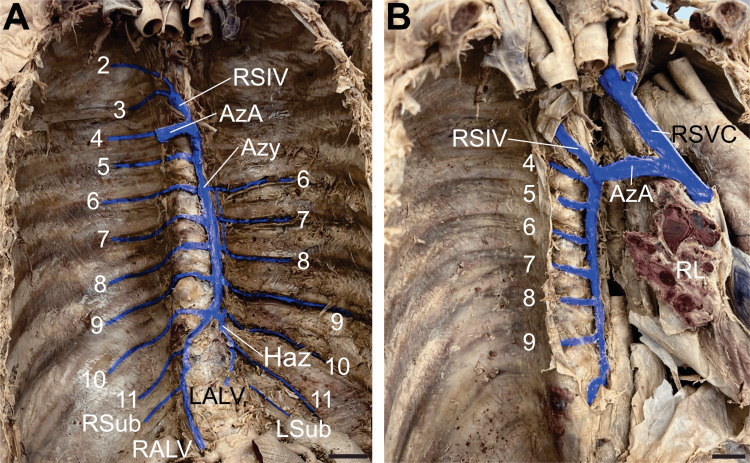
The azygos system of veins A) Anterior view of the posterior thorax showing the azygos system and its tributaries. The azygos vein (Azy) is formed by the union of the right ascending lumbar vein (RALV) and the right subcostal vein (RSub). The Azy drains right posterior intercostal veins 4-11, while right posterior intercostal veins 2-3 drain into the right superior intercostal vein (RSIV). The left ascending lumbar vein (LALV) and left subcostal vein (LSub) form an underdeveloped hemiazygos vein (Haz) draining left posterior intercostal veins 10-11. Left posterior intercostal veins 6-9 drain directly into the Azy. A small anastomosis is noted between the left posterior intercostal veins 6-8 near the Azy. The azygos arch (AzA) marks the termination of the Azy. B) Right lateral view of the thorax. This view illustrates the drainage of the Azy, RSIV, and AzA into the right superior vena cava (RSVC). Veins are digitally coloured blue. Scale bar = 2 cm.

Dissection of the posterior mediastinum and pleural cavities revealed an atypical arrangement of the azygos system. The first posterior intercostal spaces of the thorax were drained by the supreme intercostal veins bilaterally, each emptying into its respective SVC. The azygos vein was unusually positioned along the left side of the thoracic vertebral bodies (Figure 2A). On the right side, the second and third posterior intercostal veins formed the right superior intercostal vein (RSIV), which drained into the azygos arch (Figure 2A-2B). Inferiorly, the azygos vein was formed by the junction of the right subcostal and right ascending lumbar veins. It drained the posterior intercostal veins 4-11 on the right and 6-9 on the left. The azygos vein measured 127.77 mm in length and 7.36 mm in width. The left side of the thorax displayed an underdeveloped hemiazygos vein formed by the confluence of the left ascending lumbar and left subcostal veins. This small vein drained the left posterior intercostal veins 10 and 11 before joining the azygos vein at the level of the 10th rib (Figure 2A). No accessory hemiazygos vein was identified. The arch of the azygos vein crossed over the root of the right lung before joining the RSVC and measured 9.15 mm in diameter and 40.56 mm in length (Figure 2B).

Left external jugular vein variation

**Figure 3:**
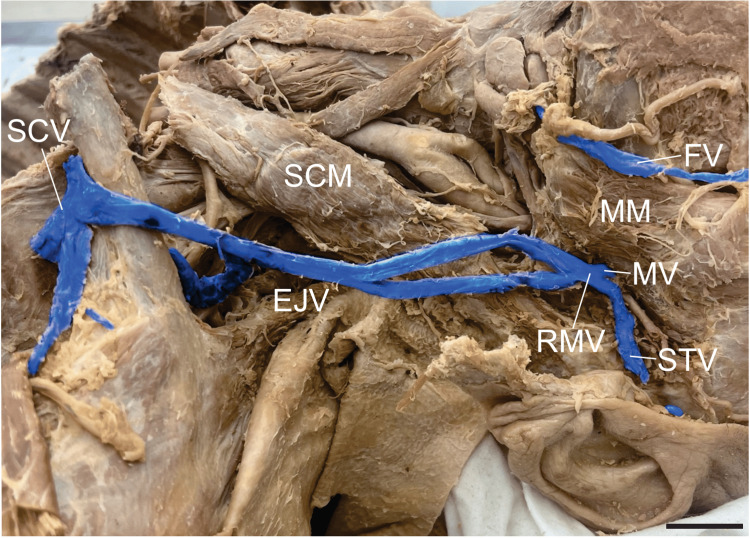
Fenestration and duplication of the left external jugular vein A lateral view of the left neck showing the facial vein (FV), masseter muscle (MM), maxillary vein (MV), superficial temporal vein (STV), retromandibular vein (RMV), sternocleidomastoid muscle (SCM), external jugular vein (EJV), and subclavian vein (SCV). The EJV exhibits a fenestration over the SCM and an inferior duplication forming a venous ring around the clavicle, with each trunk draining independently into the SCV. Veins are digitally coloured blue. Scale bar = 2 cm.

A neck dissection with parotidectomy was performed, which exposed the left EJV and its tributaries. This revealed an anatomical variation in which the maxillary and superficial temporal veins anastomosed to form a retromandibular vein, which then formed a fenestrated segment of the EJV. The two branches remained separate for 55.54 mm and rejoined at the level of the thyroid cartilage. Inferior to this point, the EJV duplicated, with the two terminal trunks spanning 33.88 mm and draining independently into the subclavian vein via a venous ring around the clavicle (Figure 3). The right EJV was unremarkable.

## Discussion

Recognizing vascular anatomical variations is critical in preventing complications during surgery, catheterization, and imaging interpretation. This report describes a rare coexistence of vascular variations, including a DSVC, MBs, an atypical configuration of the azygos system, and a fenestrated bifid EJV with a venous ring.

The persistence of the LSVC due to a failure of the left anterior cardinal vein to regress [1] presents an unusual variation in the drainage of the upper left side of the body. As the most common congenital venous anomaly of the thorax [6], awareness of this variation should hold some level of importance for most physicians and surgeons. Present in only 0.3% of the general population, DSVCs exist in 10-11% of individuals with congenital heart disease, with the variation generally being asymptomatic unless the LSVC drains directly into the left atrium [7]. However, a DSVC increases the risk of complications during CVC, cardiac transplantation, and pacemaker insertion [6]. In cases where the LSVC drains into the CS, the resulting enlargement of the CS caused by the increased blood volume is not always benign. In this case, the CS alone received venous return from the left side of the head, neck, arm, and posterior intercostal spaces 1-5 via the LSIV (Figure 1A-1C). The dilation of the CS can result in compression of elements of the conducting system of the heart, leading to arrhythmias [8,9]. Compression of the left atrium may also decrease cardiac output and complicate mitral valve surgery [10]. This case of a DSVC features two veins descending into the mediastinum without a bridging left brachiocephalic vein, a configuration reported in approximately 65% of DSVC cases [10]. With this variation, insertion of a central venous catheter through the left neck could result in cannulation of the coronary sinus, rather than the cavoatrial region. This has been associated with cases of cardiac arrhythmias, cardiogenic shock, cardiac tamponade, and CS thrombosis [6].

Inspection of the heart revealed two consecutive MBs of the LAD (Figure 1D). Myocardial bridging describes an anatomical variation in the coronary circulation where an epicardial artery tunnels into the myocardium along part of its descending path, creating a tunnelled arterial segment. Although most commonly found in the LAD, MBs can also occur in other coronary arteries [11]. During ventricular systole, MBs can occlude affected coronary artery segments and delay diastolic coronary filling, resulting in potential ischemia of the myocardium. Several studies show MBs may contribute to myocardial infarction, ventricular arrhythmias, and sudden cardiac death. The increased mechanical stress caused by vessel compression during systole, together with the structural vulnerability of the pre-bridged endothelial wall, may promote endothelial injury. Such injury can trigger platelet aggregation and provoke coronary vasospasm [12]. Symptomatic individuals may wish to undergo unroofing surgery, in which the overlying myocardium is removed, thus releasing the bridge. This procedure is thought to be a safe option for patients with isolated MBs of the LAD [13].

Figure 2 depicts an unusual azygos system within the thoracic cavity, with the azygos vein displaced and running along the left anterolateral surface of the vertebral column. To the right of the azygos vein are well-formed osteophytes, a common finding in elderly individuals. This positional change of the azygos vein with increasing age could occur due to asymmetric osteophyte growth along the vertebral column. Within the thorax, the aorta resides on the left side, potentially inhibiting left-sided osteophyte growth but not restricting right-sided osteophyte development. These osteophytes on the right side of the vertebral column could then push the azygos vein to the left side of the thorax. Interestingly, azygos veins coursing along the left side of the thorax have been observed in association with cases of persistent left superior vena cava [14]. However, given the additional congenital anomalies involving this vessel, its displacement in our case is felt to be at least partially developmental in origin. Failure to recognize such variations within the azygos vein may lead to complications such as iatrogenic intraoperative hemorrhage and radiological misdiagnoses [15]. The left side of the thorax revealed the absence of an accessory hemiazygos vein and an underdeveloped hemiazygos vein that drained only the left ascending lumbar, left subcostal, and posterior intercostal veins 10 and 11. In 2009, a similar finding was described, in which the underdeveloped hemiazygos drained the left posterior intercostal veins 8-11. This azygos system variation may be misidentified as an aneurysm, lymphadenopathy, or even a tumour on imaging [16].

The external jugular vein (EJV) in our specimen exhibited an exceptionally rare configuration characterized by a superior fenestration followed by an inferior duplication. This vein is divided into two limbs that rejoin at the level of the thyroid cartilage, forming the fenestration. Inferiorly, the vessel duplicated, creating a venous ring encircling the clavicle (Figure 3). Because the anterior and posterior limbs of this loop anastomosed independently with the subclavian vein, this configuration was classified as a true duplication rather than a second fenestration. Although previous reports have described various permutations of EJV fenestrations and duplications, this case represents, to our knowledge, a uniquely rare configuration in which a unilateral fenestration is followed by a duplication forming a venous ring around the clavicle.

Segments of the EJV crossing the clavicle anteriorly may increase the risk of injury in trauma settings. A surgical case was reported in which the EJV was found coursing anterior to the clavicle, underscoring the risks of treating patients with these unrecognized vascular variants. In the presented case, the atypical vein trajectory was encountered during open reduction of a clavicular fracture, which had to be carefully mobilized and protected to avoid intraoperative hemorrhage. The authors concluded that even seemingly routine procedures can harbour significant vascular risk when variant anatomy is present. If such anomalies are not recognized during head and neck surgery, they can lead to serious complications for the patient, particularly when sharp instruments are used without thorough visualization of the underlying anatomy [17].

## Conclusions

This case describes the coexistence of a DSVC, an atypical azygos venous arrangement, a fenestrated and duplicated left EJV with a venous ring, and double MBs, each contributing to the extensive vascular heterogeneity. Such variations are highly relevant to CVC, cardiac and thoracic surgery, and head and neck procedures, where unrecognized deviations from common vascular anatomy may precipitate iatrogenic injury. These findings reinforce the value of detailed cadaveric studies for improving surgical planning, radiologic interpretation, and medical education.

## References

[1] Sadler TW (2012). Cardiovascular system. Langman’s Medical Embryology.

[2] Marshall J (1850). On the development of the great anterior veins in man and mammalia; including an account of certain remnants of fœtal structure found in the adult, a comparative view of these great veins in the different mammalia, and an analysis of their occasional peculiarities in the human subject. Philos Trans R Soc Lond.

[3] Irwin RB, Greaves M, Schmitt M (2012). Left superior vena cava: revisited. Eur Heart J Cardiovasc Imaging.

[4] Kurtoglu E, Cakin O, Akcay S, Akturk E, Korkmaz H (2011). Persistent left superior vena cava draining into the coronary sinus: a case report. Cardiol Res.

[5] Metzler B, Hillebrand H, Eulenbruch HP, Dierkesmann R, Hust MH (2002). [Persistent left superior vena cava with right-left shunt into the left atrium]. Dtsch Med Wochenschr.

[6] Boyer R, Sidhu R, Ghandforoush A, Win T, Heidari A (2019). Persistent left superior vena cava: implications of surgical management. J Investig Med High Impact Case Rep.

[7] Zhu C, Zeitouni F, Warren L, Santos A (2022). Beware of a duplicate superior vena cava. Southwest Respir Crit Care Chron.

[8] Hsu LF, Jaïs P, Keane D (2004). Atrial fibrillation originating from persistent left superior vena cava. Circulation.

[9] Rodríguez-Mañero M, Schurmann P, Valderrábano M (2016). Ligament and vein of marshall: a therapeutic opportunity in atrial fibrillation. Heart Rhythm.

[10] Azizova A, Onder O, Arslan S, Ardali S, Hazirolan T (2020). Persistent left superior vena cava: clinical importance and differential diagnoses. Insights Imaging.

[11] Loukas M, Curry B, Bowers M (2006). The relationship of myocardial bridges to coronary artery dominance in the adult human heart. J Anat.

[12] Roberts W, Charles SM, Ang C (2021). Myocardial bridges: a meta-analysis. Clin Anat.

[13] Hemmati P, Schaff HV, Dearani JA, Daly RC, Lahr BD, Lerman A (2020). Clinical outcomes of surgical unroofing of myocardial bridging in symptomatic patients. Ann Thorac Surg.

[14] Nathan H (1960). Anatomical observations on the course of the azygos vein (vena azygos major). Thorax.

[15] Sehmi S (2022). Azygos venous system variations: a clinical review. AMEI Curr Trend Diagn Treat.

[16] Quadros LS, Potu BK, Guru A (2009). Anomalous azygos venous system in a south Indian cadaver: a case report. Cases J.

[17] Reinhardt KR, Kim HJ, Lorich DG (2011). Anomalous external jugular vein: clinical concerns in treating clavicle fractures. Arch Orthop Trauma Surg.

